# Effects of Age on Long-Term Functional Recovery in Patients with Stroke

**DOI:** 10.3390/medicina56090451

**Published:** 2020-09-07

**Authors:** Jae Wan Yoo, Bo Young Hong, Leechan Jo, Joon-Sung Kim, Jung Geun Park, Bo Kyung Shin, Seong Hoon Lim

**Affiliations:** Department of Rehabilitation Medicine, St. Vincent’s Hospital, College of Medicine, The Catholic University of Korea, Seoul 06591, Korea; ainshu@gmail.com (J.W.Y.); byhong@catholic.ac.kr (B.Y.H.); joychan85@hotmail.com (L.J.); svpmr@chol.com (J.-S.K.); jgp0123@naver.com (J.G.P.); bogyeong0412@gmail.com (B.K.S.)

**Keywords:** stroke, functional recovery, outcome, age effect

## Abstract

*Background and objectives:* Age might be a determinant that limits functional recovery in patients with stroke. Here, we investigated the effect of age on functional recovery within 30 months after stroke onset. *Materials and Methods:* This retrospective longitudinal study enrolled 111 patients with first-ever stroke. Functional recovery was assessed at 2 weeks and at 1, 6, and 30 months after stroke onset using the modified Barthel Index (MBI), modified Rankin Score (mRS), functional ambulation category (FAC), muscle strength, and Mini-Mental State Examination (MMSE). A generalized estimating equation analysis was performed. *Results:* With the MBI, function improved until 6 months after stroke onset in patients aged <70 years and until 1 month after stroke onset in patients ≥70 years. At 30 months after stroke, there was no significant change of MBI in patients aged <70 years, whereas there was a significant decline in older patients. With the mRS and FAC, function improved until 30 months after stroke onset in patients aged <70 years and until 1 month after stroke onset in older patients. Motor deficit, assessed using the Medical Research Council (MRC), improved significantly until 6 months after stroke onset in patients aged <70 years. There was a significant improvement in cognition (assessed using the MMSE) until 6 months after stroke onset in patients aged <70 years and until 1 month after stroke onset in older patients. *Conclusions:* Long-term functional recovery occurred for up to 30 months after stroke. Patients aged ≥70 years showed functional decline between 6 and 30 months after onset. These findings could be useful when measuring functional recovery after stroke.

## 1. Introduction

Stroke is a leading cause of disability and death worldwide; patients with stroke may need some assistance or be fully dependent on family or caregivers for activities of daily living (ADL) [1,2]. Functional recovery of ADL continues for at least 6 months after stroke onset [3,4] and is related to various factors, including age [5,6], sex [7,8], stroke event [9,10], motor impairment [11,12], and cognitive dysfunction [13,14]. Using the Barthel Index to study functional recovery for up to 3 years in 39 patients with stroke, Johnston et al. found that function improved within 12 months after stroke onset and was maintained between 12 and 36 months after stroke onset [15]. However, they did not examine the effect of age on functional recovery. Another study using the modified Rankin Scale (mRS) did not compare functional recovery between age groups [16]. A third study found a negative effect of age on functional recovery in univariate analysis; however, it did not uncover a specific age for functional decline [17]. However, animal study results revealed age may be a barrier for plasticity [18,19].

Assessment of clinical outcome and early prediction of clinical recovery are very important for patients with stroke and their clinicians. We hypothesized that age influences long-term functional recovery in patients with stroke. Therefore, this study investigated the effects of age on ADL and the specific age at which a long-term functional decline occurs for up to 30 months after stroke onset.

## 2. Materials and Methods

### 2.1. Study Design

This retrospective longitudinal observational study recruited 111 patients with first-ever stroke from a single center between January 2011 and September 2019. All patients had ischemic or hemorrhagic stroke and met the following criteria: (1) 20–89 years of age; (2) stroke diagnosis by computed tomography or magnetic resonance imaging; and (3) initial assessment within 2 weeks after onset, subsequent assessment at 1 or 6 months after onset, and final assessment approximately 2 years after onset. Exclusion criteria were as follows: (1) any symptomatic brain disorders other than first-ever stroke and (2) any other medical disorder resulting in pre-mobility of non-independent gait and ADL. All patients received physical (neurodevelopmental treatment approach) and occupational (task-orientated approach) therapies. All rehabilitation programs began within 5 days after onset. Treatment continued until 6 months after onset and consisted of both physical and occupational therapy for 1–2 h per day, 5 days per week. Speech therapy was provided as needed. Interventions focused on use and strengthening of affected limbs, basic mat activities, symmetric weight-bearing and transfer activities, and gait training; interventions were not performed exclusively for a particular purpose. All patients had their demographic data recorded, underwent computed tomography or high-resolution magnetic resonance imaging to examine brain lesions within 30 days after onset, and underwent functional evaluation, including assessments of ADL, motor function, ambulation, and cognition. The study protocol was reviewed and approved by the Institutional Review Board of the Catholic University, College of Medicine (Registry No. VC19RESI0207) on 18 October 2019, which waived the requirement for informed consent.

### 2.2. Assessment

Clinical assessments were conducted at baseline (within 2 weeks of stroke onset) and at 1 and 6 months after stroke onset. Final assessments were performed between 30 and 36 months after stroke onset. Functional disability was assessed using the Korean version of the modified Barthel Index (MBI) [20], which consists of 10 subscales with scores ranging from 0 (completely dependent) to 100 (independent in basic ADL) as the primary outcome [21]. The functional ambulation category (FAC) [22] and modified Rankin Scale (mRS) [23] were assessed as other functional outcomes. To assess motor deficit recovery, the Medical Research Council (MRC) scale [24] was used; the MRC scales for elbow flexion and wrist flexion were summed to determine motor function of the affected arm, while those for hip extension and knee extension were summed to determine motor function of the affected leg. Recovery of cognition was measured by the Korean version of the Mini-Mental State Examination (MMSE) [25].

### 2.3. Statistical Analysis

Statistical analyses were performed using IBM SPSS Statistics, version 21.0 (IBM Corp., Armonk, NY, USA). Patients were stratified into four groups according to age (<50, 50–59, 60–69, and ≥70 years) to examine the effects of age, and into two groups (20–69 and 70–89 years) to focus on the effect [26,27,28]. The patients’ demographic and clinical characteristics were compared using one-way analysis of variance, the Kruskal–Wallis test for continuous variables, and the chi-squared test for categorical variables; these comparisons were performed after the normalities of variable distributions had been evaluated using the Shapiro–Wilk test. All assessments compared groups at 2 weeks, 1 month, 6 months, and 30 months, using generalized estimating equation analysis; these comparisons allowed inclusion of the data of patients with missing variables. Two fixed effects were included in the model: the within-patient time effect (2 weeks, 1 month, 6 months, and 30 months) and between-patients group effect. If the effect identified using generalized estimating equation analysis was statistically significant (*p* < 0.05), a pairwise comparison was performed using the estimated marginal means to identify the two measurement points at which a significant difference occurred. Post hoc analysis with the Bonferroni method was used; for multiple comparisons, *n* was set at *p* < (0.05/*n*).

## 3. Results

**Figure 1:**
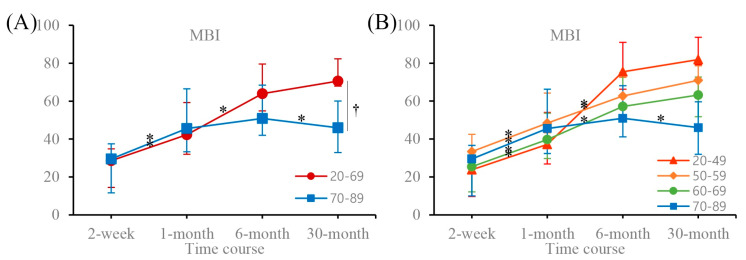
Time course of MBI scores for (**A**) two and (**B**) four age groups at 2 weeks, 1 month, 6 months, and 30 months. Data are expressed as the mean (95% confidence interval). MBI, Modified Barthel Index. MBI scores increased significantly within 6 months after stroke onset and did not change significantly between 6 and 30 months after stroke onset in patients aged <70 years. Patients aged ≥70 years exhibited significant increases in MBI scores until 1 month after stroke onset and a decline between 6 and 30 months after stroke onset. * Adjusted *p*-value < 0.05 for within-patient time effect; pairwise comparison with former score for time. † Adjusted *p*-value < 0.05 for between-patients group effect; pairwise comparison with adjacent score for age group.

**Figure 2:**
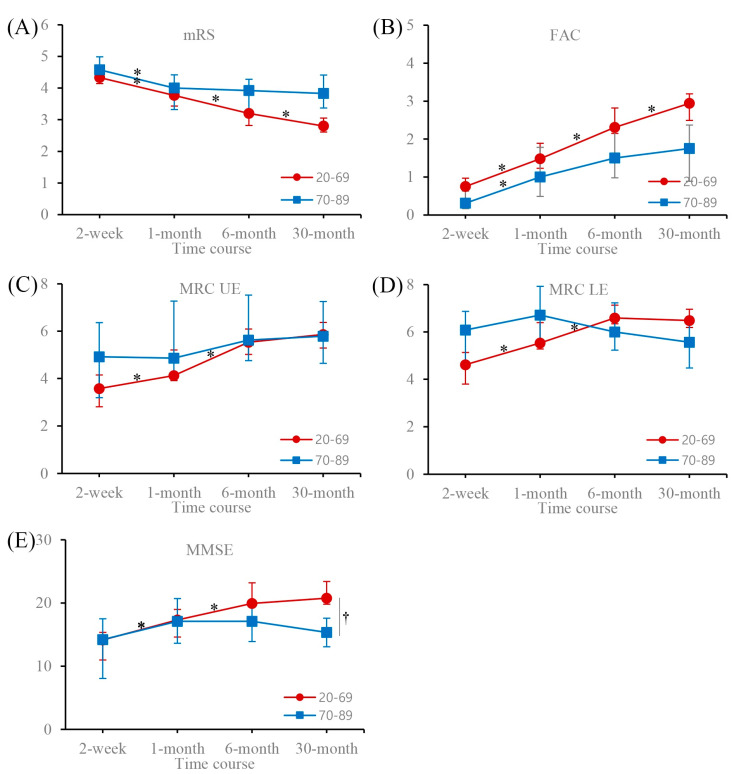
Time course of functional assessment scores for two age groups at 2 weeks, 1 month, 6 months, and 30 months. Data are expressed as the mean (95% confidence interval). (**A**) mRS, modified Rankin Scale; (**B**) FAC, functional ambulation category; (**C**) MRC_UF_, sum of MRC scores for elbow and wrist flexion of the affected arm; (**D**) MRC_LE_, sum of MRC scores for hip and knee extension of the affected leg; (**E**) MMSE, Mini-Mental State Examination. mRS and FAC scores continued to increase significantly until 30 months after stroke onset in patients aged <70 years, and until 1 month after stroke onset in older patients. There was no significant change between 1 and 30 months after stroke onset in patients aged ≥70 years. MRC scores of the affected upper and lower extremities showed that motor deficit improved significantly until 6 months after stroke onset and did not change significantly between 6 and 30 months after stroke onset in patients aged <70 years. No significant changes in MRC scores were observed at any time in older patients. MMSE scores increased significantly until 6 months after stroke onset in patients aged <70 years, and until 1 month after stroke onset in older patients. There were no significant changes at any other time. * Adjusted *p*-value < 0.05 for within-patient time effect; pairwise comparison with former score for time. † Adjusted *p*-value < 0.05 for between-patients group effect; pairwise comparison with adjacent score for age group.

Table 1 presents the patients’ demographic and clinical characteristics. In patients aged <70 years, the MBI significantly increased until 6 months after stroke onset and did not change significantly between 6 and 30 months. In patients aged ≥70 years, there was a significant increase in MBI until 1 month after stroke onset and a decline between 6 and 30 months after stroke onset. A between-patients group effect was observed at 30 months (Figure 1, Table 2). The results for four age groups (20–49, 50–59, 60–69, and 70–89 years) were the same as those for two age groups (20–69 and 70–89 years; Figure 1, Table 3). The mRS and FAC continued to increase significantly until 30 months after stroke onset in patients aged <70 years, and until 1 month after stroke onset in patients aged ≥70 years. There were no significant changes between 1 and 30 months after stroke onset in older patients (Figure 1, Table 2). The MRC scores of the affected upper and lower extremities improved significantly until 6 months after stroke onset and did not change significantly between 6 and 30 months after stroke onset in patients aged <70 years. No significant change in MRC scores was observed at any time in patients aged ≥70 years (Figure 2, Table 2). The MMSE scores increased significantly until 6 months after stroke onset in patients aged <70 years and until 1 month after stroke onset in patients aged ≥70 years. There were no significant changes at any other time (Figure 2, Table 2).

## 4. Discussion

We investigated the effect of age on functional recovery using the MBI until 30 months after stroke onset. All parameters for impairment, disability, and handicap via MBI, mRS, FAC, MMSE, and motor strength improved significantly until 6 months after stroke onset in patients aged less than seventy. Based on the mRS and FAC, the functional recovery continued to improve significantly until 30 months after stroke onset in patients aged less than seventy years. The functional recovery for older subjects than seventy continued to 1 month after stroke onset and reached a plateau for mRS, FAC, MMSE, and muscle strength up to 30 months after stroke onset. The score of MBI for subjects ≥ 70 was declined between 6 and 30 months after onset. Therefore, age ≥70 years might limit long-term functional recovery in patients with stroke.

The previous research showed the motor strength had usually improved within 3 to 6 months after stroke onset [12,29,30]. In consistent with these studies, our results revealed that the muscle strength significantly improved up to 6 months after onset in patients aged less than 70 years. However, the muscle strength of patients ≥ 70 did not significantly improved since 1 month after onset. The MMSE showed that cognitive dysfunction improved significantly until 6 months after stroke onset in patients aged <70 years and until 1 month after stroke onset in older patients. Similarly to the findings regarding motor function, there was no improvement in cognition beyond 6 months after stroke onset at all ages.

In our patients, recovery of motor and cognition beyond 6 months after stroke onset did not differ according to age; however, functional recovery at 6 to 30 months differed between patients aged <70 years and those aged ≥70 years. A previous study found that functional recovery occurred to a greater extent in younger patients within 3 months after stroke onset [16]. In another study, functional recovery was maintained within 12 months after stroke onset [31]. Our results are consistent with the findings of previous studies. Many prior studies showed that functional recovery until 6 months after stroke onset was better in younger patients and then persisted until 30 months after stroke onset in all patients; conversely, we observed a functional decline between 6 and 30 months after stroke onset in patients aged ≥70 years. In animal studies, the neuronal sprouting, neurogenesis, and angiogenesis were limited in aged mice as 24 months [18,19]. Taken together, age effects on neural plasticity may be occurred universally in mammals.

This study had several limitations. First, we needed to enroll over 100 patients using a longitudinal approach to minimize bias due to the retrospective design of the study. Second, we did not examine more detailed time points between 6 and 30 months after stroke onset. Finally, we do not know the cause of the functional decline in patients aged ≥70 years. Vascular dementia may limit the ADL of older patients. Musculoskeletal disorders (e.g., osteoarthritis or nursing care unit factors) may also have contributed to the findings. We excluded cognitive decline as a source of functional decline in older patients by confirming that there was no change in the MMSE score; however, we could not rule out other causes.

## 5. Conclusions

Our results show a long-term pattern of functional recovery with age for up to 30 months after stroke onset. In patients aged <70 years, functional improvement was observed until 6 months after stroke onset; this was maintained from 6 to 30 months after stroke onset. In contrast, older patients exhibited functional decline between 6 and 30 months after stroke onset. These findings could be useful when measuring functional recovery in patients with stroke, as well as for development of practical treatment plans for older patients.

## Figures and Tables

**Table 1:** Patient demographics and clinical characteristics.

	Total (*n* = 111)
Age, years	58.2 ± 11.5
Female sex	52 (46.8)
Stroke type	
Ischemic	61 (55.0)
Hemorrhagic	50 (45.0)
Lesion side, left side	65 (58.6)
Lesion location ^a^	
Cortical	51 (45.9)
Subcortical	62 (55.9)
Brainstem	10 (9.0)
Cerebellum	9 (8.1)
Coexisting conditions ^a^	
Atrial fibrillation	10 (9.0)
Hypertension	54 (48.6)
Diabetes mellitus	22 (19.8)
Dyslipidemia	32 (29.4)
Smoking history	31 (27.9)
Alcohol history	46 (41.4)

Data are expressed as the mean ± standard deviation or *n* (%). ^a^ Multiple counts were allowed, including all lesions involved.

**Table 2:** Time course of assessment scores for two age groups.

	Age	Time Course			
		2 Weeks	1 Month	6 Months	30 Months
MBI	20–69	28.59 (20.31–32.24)	42.33 * (38.77–52.75)	63.98 * (60.99–73.29)	70.58 (65.54–76.62)
	70–89	29.50 (11.61–37.44)	45.57 * (33.32–66.51)	50.91 (41.97–68.40)	46.00 *^,^† (32.84–60.03)
mRS	20–69	4.33 (4.14–4.59)	3.77 * (3.43–3.92)	3.20 * (2.82–3.28)	2.80 * (2.61–3.05)
	70–89	4.58 (4.30–4.99)	4.00 * (3.32–4.42)	3.92 (3.31–4.28)	3.83 † (3.37–4.41)
FAC	20–69	0.75 (0.38–0.97)	1.48 * (1.23–1.89)	2.31 * (2.15–2.82)	2.94 * (2.49–3.19)
	70–89	0.31 (0.17–0.63)	1.00 * (0.49–1.78)	1.50 (0.98–2.33)	1.75 (0.89–2.37)
MRC_UF_	20–69	3.58 (2.81–4.15)	4.13 * (3.91–5.21)	5.53 * (5.02–6.09)	5.86 (5.29–6.37)
	70–89	4.92 (3.19–6.36)	4.86 (4.27–7.27)	5.62 (4.76–7.52)	5.79 (4.64–7.25)
MRC_LE_	20–69	4.62 (3.80–5.13)	5.53 * (5.28–6.39)	6.59 * (6.34–7.13)	6.48 (6.19–6.96)
	70–89	6.08 (4.72–6.87)	6.71 (5.53–7.92)	6.00 (5.23–7.23)	5.57 (4.48–6.65)
MMSE	20–69	14.15 (10.98–15.34)	17.33 * (14.62–18.98)	19.93 * (19.40–23.20)	20.76 (19.81–23.40)
	70–89	14.22 (8.06–17.49)	17.11 * (13.64–20.71)	17.10 (13.92–20.12)	15.36 † (13.07–17.61)

Data are expressed as the mean (95% confidence interval). MBI, Modified Barthel Index; mRS, modified Rankin Scale; FAC, functional ambulation category; MRC_UF_, sum of MRC scores for elbow and wrist flexion of affected arm; MRC_LE_, sum of MRC scores for hip and knee extension of affected leg; MMSE, Mini-Mental State Examination. All functional scores were compared between two age groups at 2 weeks, 1 month, 6 months, and 30 months, using generalized estimated equation analysis. Pairwise comparison was performed using estimated marginal means to identify a within-patient time effect or between-patients group effect. The *p*-value was adjusted based on Bonferroni correction for multiple comparisons in post hoc analysis. * Adjusted *p*-value < 0.05 for within-patient time effect; pairwise comparison with former score for time. † Adjusted *p*-value < 0.05 for between-patients group effect; pairwise comparison with adjacent score for age group.

**Table 3:** Time course of MBI scores for four age groups.

	Age	Time Course			
		2 Weeks	1 Month	6 Months	30 Months
MBI	20–49	23.75 (9.65–30.00)	37.17 * (26.83–54.04)	75.45 * (66.28–91.03)	81.91 (79.26–93.61)
	50–59	33.37 (22.8–42.42)	48.37 * (43.30–64.25)	62.65 * (55.63–72.51)	70.92 (62.39–78.65)
	60–69	25.36 (12.12–32.67)	39.70 * (29.66–53.56)	57.14 * (50.57–75.06)	63.17 (51.76–72.70)
	70–89	29.50 (9.97–36.68)	45.57 * (32.32–66.27)	50.91 (41.14–68.15)	46.00 * (31.96–59.60)

Data are expressed as mean (95% confidence interval). MBI, Modified Barthel Index. K-MBI scores were compared among four age groups at 2 weeks, 1 month, 6 months, and 30 months, using generalized estimated equation analysis. Pairwise comparison was performed using estimated marginal means to identify a within-patient time effect or between-patients group effect. The *p*-value was adjusted based on Bonferroni correction for multiple comparisons in post hoc analysis. * Adjusted *p*-value < 0.05 for within-patient time effect; pairwise comparison with former score for time.

## Abbreviations

MBI	modified Barthel Index
mRS	modified Rankin Score
FAC	functional ambulation category
MMSE	Mini-Mental State Examination
